# Humanized Mouse Models of Systemic Lupus Erythematosus: Opportunities and Challenges

**DOI:** 10.3389/fimmu.2021.816956

**Published:** 2022-01-18

**Authors:** Jiaxuan Chen, Shuzhen Liao, Huimin Zhou, Lawei Yang, Fengbiao Guo, Shuxian Chen, Aifen Li, Quanren Pan, Chen Yang, Hua-feng Liu, Qingjun Pan

**Affiliations:** ^1^ Key Laboratory of Prevention and Management of Chronic Kidney Disease of Zhanjiang City, Institute of Nephrology, Affiliated Hospital of Guangdong Medical University, Zhanjiang, China; ^2^ Zhanjiang Central Hospital, Guangdong Medical University, Zhanjiang, China

**Keywords:** systemic lupus erythematosus, immunodeficient mouse, humanized SLE mouse, autoantibodies, pro-inflammatory cytokines, lupus nephritis

## Abstract

Animal models have played a crucial role in the understanding of the mechanisms and treatments of human diseases; however, owing to the large differences in genetic background and disease-specific characteristics, animal models cannot fully simulate the occurrence and progression of human diseases. Recently, humanized immune system mice, based on immunodeficient mice, have been developed that allow for the partial reconstruction of the human immune system and mimic the human *in vivo* microenvironment. Systemic lupus erythematosus (SLE) is a complex disease characterized by the loss of tolerance to autoantigens, overproduction of autoantibodies, and inflammation in multiple organ systems. The detailed immunological events that trigger the onset of clinical manifestations in patients with SLE are still not well known. Two methods have been adopted for the development of humanized SLE mice. They include transferring peripheral blood mononuclear cells from patients with SLE to immunodeficient mice or transferring human hematopoietic stem cells to immunodeficient mice followed by intraperitoneal injection with pristane to induce lupus. However, there are still several challenges to be overcome, such as how to improve the efficiency of reconstruction of the human B cell immune response, how to extend the lifespan and improve the survival rate of mice to extend the observation period, and how to improve the development of standardized commercialized models and use them. In summary, there are opportunities and challenges for the development of humanized mouse models of SLE, which will provide novel strategies for understanding the mechanisms and treatments of SLE.

## Introduction

**Figure 1:**
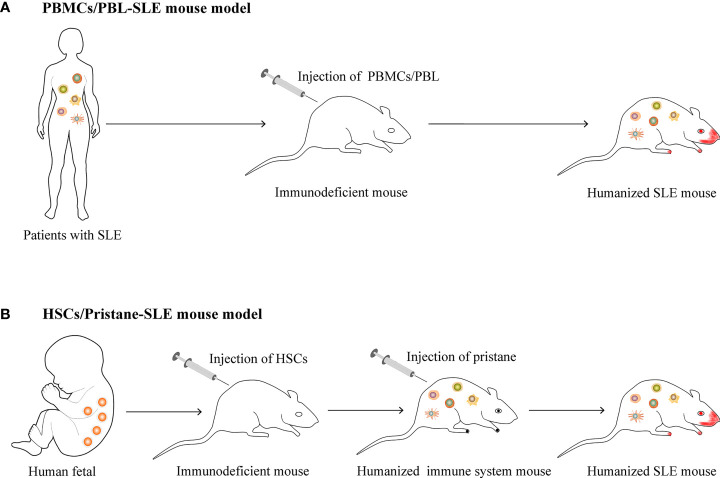
The construction of humanized SLE mouse model. **(A)** Transferring human peripheral blood mononuclear cells (PBMCs) or peripheral blood lymphocytes (PBLs) from patients with SLE to immunodeficient mice. **(B)** Transferring human hematopoietic stem cells (HSCs) to immunodeficient mice and then injecting intraperitoneally (*i. p.*) with pristane to induce lupus.

Systemic lupus erythematosus (SLE) is a typical autoimmune disease characterized by excessive activation of T and B cells, producing a large number of autoantibodies and pro-inflammatory cytokines that result in tissue and organ damage (1). At present, there are few clinically approved traditional therapeutic drugs and biologic therapies for SLE (2, 3). Animal models have made great contributions to the study of SLE pathogenesis and the development of new drugs. Based on the study of spontaneous (4–6) or induced (7–9) lupus-prone mouse model, considerable progress has been made in understanding the pathogenesis of SLE. In these models, disease phenotypes similar to patients with SLE can be observed, including the imbalanced immune responses of T and B cells, the production of a variety of autoantibodies and a large number of pro-inflammatory cytokines, and damage to multiple organs (such as lupus nephritis, etc.) (10). However, the genetic background differences between humans and mice cause the lupus-prone mouse model to have many differences from human SLE, especially when studying the *in vivo* functions of molecules with poor homology between humans and mice (such as non-coding RNA, etc.) (11–13) and Kv1.3 phenotype, etc. (14). The emergence of humanized mice allows for better studies *in vivo*, further clarifies the pathogenesis, and improves the success rate of translational medicine research (such as novel drug discovery, etc.) (15–17). At present, there are two main methods of constructing humanized mouse models of SLE, including transferring human peripheral blood mononuclear cells (PBMCs) or peripheral blood lymphocytes (PBLs) from patients with SLE to immunodeficient mice (18, 19), or transferring human hematopoietic stem cells (HSCs) to immunodeficient mice and then injecting intraperitoneally (*i. p.)* with pristane to induce lupus (20) (**Figure 1**). For these two humanized SLE mouse models, the PBLs/PBMCs humanized mouse model is widely used, but individual differences in SLE patients often lead to inconsistent model parameters and poor uniformity; the HSCs-pristane humanized mouse model can better reproduce the clinical features of human SLE, but there are very few such studies. The differences between two kinds of humanized SLE mice as shown in **Table 1**. The above two humanized SLE mouse models provide opportunities to study the pathogenesis and prevention of SLE *in vivo*, but there are also many challenges.

**Table 1:** The differences between two kinds of humanized SLE mice.

	PBLs/PBMCs humanized mouse model	HSCs-pristane humanized mouse model
Methods	PBLs/PBMCs from patients with SLE were injected intravenously or intraperitoneally into immunodeficient mice	Human HSCs were injected intravenously into immunodeficient mice followed by pristane intraperitoneally
Immune cells	Human CD45^+^ cells accounted for 20–80% of peripheral blood (21)	Human T cells, B cells, and NK cells in peripheral blood of mice ↓ (20)
	Human CD4^+^ T cells ↓, CD8^+^ T cells ↑ in peripheral blood (21, 22)	Human CD19^+^ CD20^-^ CD27^hi^ CD38^hi^ plasmablasts/plasma in peripheral blood and spleen of mice ↑ (20)
	Human IL-17^+^ Tfh cells in spleen ↑ (23)	Human CD27^+^ memory B cells and CD2^7-^ IgD^-^ B cells in peripheral blood and spleen of mice ↑ (20)
		Human CD27^-^ IgD^+^ naïve/transitional B cells in peripheral blood and spleen of mice ↓ (20)
Auto-antibodies	Human IgG ↑ (24, 25)	Human anti-nuclear autoantibodies (anti-dsDNA, anti-histone, anti-RNP70, anti-SM, anti-SSA IgGs) ↑ (20)
	Human IgG I ↑ (22)	
	Human IgG II ↑ (26)	
	Human IgG, IgA, IgM ↑ (27)	
	Human anti-dsDNA↑ and mostly IgG I, IgG II (28)	
	Human anti-dsDNA ↑ (16, 17, 23, 29–33)	
	Human anti-Ro, anti-La, anti-RNP ↑ (34)	
	Human anti-ssDNA, anti-RNA, anti-histone, anti-nucleosome ↑ (15)	
Pro-inflammatory cytokines	Human IL-10 ↑ (27)	Human IFN-γ, IL-6, IL-8, IL-18, MCP-1 ↑ (20)
	Human IFN-γ, IL-4 ↑ (15)	
	Human IFN-γ, IL-10 ↑ (16, 31)	
	Human IFN-γ, IL-10 ↑, TGF-β ↓ (29)	
Renal function	Proteinuria ↑ (14)	Proteinuria ↑ (20)
	Proteinuria ↑ and human IgG deposition in glomeruli (15, 21, 23, 32)	Human CD45^+^ cells, IgG, and IgM deposition in glomeruli (20)
	Human IgG, IgA deposition in glomeruli (22)	
	Human IgG, IgA, IgM deposition in glomeruli (17)	
	Human IgG, IL-17A deposition in glomeruli (30)	
	Human IgG, C3 deposition in glomeruli (29)	
Survival rates	Survival rates ↓ (14, 21, 25, 26)	Median survival at 13 weeks (20)

“↑/↓” in PBLs/PBMCs humanized mouse models represent an increase or decrease compared to healthy PBLs/PBMCs controls.

“↑/↓” in HSCs-pristane humanized mouse models represent an increase or decrease compared to no pristane controls.

## PBLs/PBMCs Humanized SLE Mouse Model

### Development of PBLs/PBMCs Humanized SLE Mouse Model

The main characteristic of humanized mice is the reconstruction of the human immune system in immunodeficient mice. For DKO (BALB-Rag2^-/-^ IL2Rgc^-/-^) mice (4–5 weeks old) engrafted with PBMCs (0.3–0.5×10^7^) from patients with SLE, the ratio of human CD45^+^ cells to total PBMCs increased from 5–10% (6–7 weeks old) to 20–80% (8–10 weeks old) (21).

It is known that T and B cells interact to promote the progression of lupus (35). T cells mainly promote the development of SLE through the production of pro-inflammatory cytokines and tissue infiltration (36). Humanized mouse models of SLE constructed by engrafting PBLs/PBMCs from patients with SLE have mainly revealed the presence of human T cells (21). A skewed ratio of CD4 to CD8 (lower frequency of CD4^+^ and higher CD8^+^ cells) in the PBMCs of patients with SLE is commonly observed (37, 38). In a humanized mouse model of SLE within 7–8 weeks by engrafting PBMCs (0.3–0.5×10^7^) from patients with SLE, CD3^+^ cells were found in the CD45^+^cells of PBMCs from both SLE-DKO and ND-DKO mice. While in SLE-DKO mice, a significantly lower frequency of CD3^+^CD4^+^cells (5.5% ± 2.1%) and a higher frequency of CD3^+^CD8^+^ cells (79.4 ± 3.6%) was reported; this contrasted with a more typical distribution of CD3^+^CD4^+^ (66.2 ± 2.5%) and CD3^+^CD8^+^ (16.5 ± 2.1%) cells in the ND-DKO mice (21). In addition, a similar study showed that among T cells, the ratio of CD4^+^CD8^-^ cells to CD4^-^CD8^+^cells were 3:1, and 1:2 at one- and two-months post engraftment, respectively (22). This skewed distribution could also be detected in humanized SLE mice, which supports the hypothesis that this model mimics the characteristics of human SLE.

Previous studies have demonstrated that the effective B cell helper activity of Th17 cells was an important function of pro-inflammatory T cells (39, 40). The increased percentage of human IL-17^+^ Tfh cells was detected in the spleens of NSG mice (8 weeks old) engrafted with PBMCs (1×10^7^cells/mouse) from patients with active lupus, while this process could be halted by the knockdown of RORγ in human CD4^+^ T cells (23). In this instance, Th17 could also be detected in humanized SLE mice, and RORγ therapy targeting CD4^+^ T cells is expected to become a novel strategy.

In SLE, B cells mainly play a role in antibody production, antigen presentation, and cytokine expression (41). NK cells are producers of various cytokines and chemokines (e.g., IFN-g, TNF-a, CCL5, CCL3, and CCL4), which amplify and recruit an inflammatory response through various mechanisms, further contributing to the progression of SLE (42). Myeloid cells (e.g., neutrophils, dendritic cells) have also been reported to be key factors in SLE (43, 44). However, the reconstruction efficiency of B, NK, and myeloid cells in the PBLs/PBMCs humanized SLE mouse model is not satisfactory and remains a huge challenge today. In this mice model, it was found that human CD3^+^ populations were detected in the CD45^+^cells in PBMCs of SLE-DKO mice at 3–4 weeks post engraftment, while other human immune cells such as B cells, NK cells, and myeloid cells were rare or undetectable (21). Therefore, improving the reconstruction efficiency of B cells, NK cells, and myeloid cells is a significant challenge to develop this model better.

The success of reconstruction in humanized SLE mice with engraftment of human PBLs/PBMCs can also be assessed based on the expression of human IgG, with successful engraftment indicated by equal to or higher than 200 µg/mL of human IgG in the sera two weeks following PBLs/PBMC administration (28). As previously reported, the average serum level of human IgG was approximately 3000 µg/mL post-PBLs (3×10^7^ cells/mouse) from patients with SLE were injected *i.p.* into SCID mice (8–10 weeks old) (28). A similar study showed that approximately 500 µg/mL of human IgG in the serum of SCID mice (9–13 weeks old) engrafted with PBMCs (3×10^7^ cells/mouse) from patients with SLE could be detected (29).

The ratio of human CD4/CD8 also can affect the production of IgG in humanized SLE mice. When this ratio increased from a lower ratio (less than 0.5) to a higher ratio (greater than 1.5), serum levels of human IgG could be detected (24). In addition, *in vitro* activation of human PBMCs also led to ten times higher IgG production *in vivo* compared with PBMCs without activation (24). Therefore, for the variation in human IgG production in humanized mice, the key effector may be the phenotype and activation status of human PBLs/PBMCs.

For the autoantibodies produced by this PBLs/PBMCs humanized SLE mouse model, several main autoantigens (e.g., dsDNA, Ro, RNP, anti-La, etc.), and subtypes of IgGs (e.g., IgG I, IgG II) can be detected. Human IgG could be detected in the serum of established humanized SLE mice at two weeks and reached maximum levels at two months after the reconstruction of the model with *i.p.* injection of PBLs (1.5×10^7^ cells/mouse) from patients with SLE into SCID mice (22).

Anti-dsDNA autoantibodies are representative autoantibodies for the diagnosis and modeling of the disease activity of SLE (45). A previous study showed that higher levels of human IgG I and IgG II anti-dsDNA autoantibodies were detected in the serum of humanized SLE mice (10–12 weeks old), after the establishment of this model with *i.p.* injection of PBMCs (3×10^7^ cells/mouse) from patients with SLE (SLEDAI score 5.88 ± 4.18) into SCID mice (8–10 weeks) (28).

In addition, humanized SLE mice produce autoantibodies against other autoantigens. After the establishment of humanized SLE mice with *i.p.* injection of 2–5×10^7^ PBMCs from patients with SLE into SCID mice (5–7 weeks old), antibodies against human anti-Ro, anti-RNP, and anti-La in serum could be detected at 4–6 weeks after transplantation (34). DKO mice (4–5 weeks old) were used for engraftment of (0.3–0.5×10^7^ cells/mouse) PBMCs from patients with SLE, and the antibodies of humans (e.g., anti-dsDNA antibody, ANA antibodies, ACL IgG) in serum could be detected at 4–8 weeks post engraftment. Importantly, SLE patients with a high level of antiphospholipid antibodies (>80 GCL) showed high ACL IgG levels in all DKO mice engrafted with their PBMCs. Additionally, all mice had detectable ACL IgG at two to three different times within two to four weeks post engraftment (21). Thus, the engrafted mice showed most of the antibodies in patients with SLE and reflected an accurate phenocopy of certain autoantibodies.

### Treatment of PBLs/PBMCs Humanized SLE Mouse Model

#### Autoantibodies and Pro-Inflammatory Cytokines

In terms of intervention studies, the production of autoantibodies and pro-inflammatory cytokines in humanized SLE mice can also be attenuated or eliminated by drugs or other factors.

The protein annexin A1 (ANX A1) is a modulator of the immune response involving several cell types, and its expression in activated B and T cells is abnormal in autoimmune disease (46–48). In one study, the levels of autoantibodies s (e.g., anti-ssDNA, anti-RNA, anti-histone, and anti-nucleosome IgG), inflammatory cytokines (e.g., IFN-γ and IL-4), and disease symptoms were significantly reduced in anti-ANX A1 antibody-treated humanized SLE mice (12 weeks old NSG mice engrafted with 1×10^7^ PBMCs/mouse from patients with SLE), compared to the humanized SLE mice treated with the isotype control antibody (15).

Myeloid-derived suppressor cells (MDSCs) with immunosuppressive functions are a group of highly heterogeneous populations derived from myeloid progenitors (49). It has been reported that MDSCs have a pathogenic role in promoting the development of autoimmune diseases (50–52). For example, mouse MDSCs can promote the differentiation of Th17 cells (53). However, the role of MDSCs in Th17 differentiation and the pathogenesis of autoimmune diseases in humans is relatively unknown. In a previous study, a humanized SLE mouse model was established by intravenous injection of PBMCs from patients with active SLE into immunodeficient non-obese diabetic/severe combined immunodeficient (NOD/SCID) mice. To investigate the function of MDSCs and Arg-1 in disease progression, the NOD/SCID mice were injected with unaltered PBMCs, MDSC-depleted PBMCs, or unaltered PBMCs plus nor-NOHA (the Arg-1 inhibitor). The study showed that all mice (4–5 weeks old NOD/SCID mice engrafted with 0.5–1×10^7^ PBMCs/mouse from patients with SLE) injected with unaltered PBMCs had detectable human autoantibodies within four to five weeks (30). However, mice receiving MDSC-depleted PBMCs showed significantly less severe symptoms, indicating that MDSCs are necessary for disease progression *in vivo*. In addition, the deleterious role of MDSCs was possibly dependent on Arg-1, because its inhibitor significantly delayed disease progression in NOD/SCID mice (30). The above research indicates that targeting MDSCs or Arg-1 is expected to alleviate SLE disease progression.

Based on the suppressive activity of complement receptor type 1 on human lymphocytes, the co-crosslinking of this receptor on B cells with the B-cell receptor (BCR) can inhibit the activation and proliferation of B cells, and this receptor may be a novel therapeutic target for negative signal delivery (54, 55). Humanized SLE mice (8 weeks old SCID mice engrafted with 1×10^7^ PBMCs/mouse from patients with SLE) were treated with anti-human DNA-like chimeras, which contained a monoclonal antibody against human inhibitory complement receptor type 1. The results showed that anti-dsDNA antibodies were directly eliminated. The specific clearance of autoreactive B cells not only limited the production of anti-dsDNA IgG, but also limited the activation and proliferation of autoreactive T cells. Additionally, the levels of pro-inflammatory cytokines IL-10 and IFN-γ were also reduced (16, 31). The same study showed that anti-human DNA-like chimeras could prevent the production of anti-dsDNA IgG antibodies (32). Anti-human DNA-like chimeras had an ideal therapeutic effect in humanized SLE mice, and they are expected to enter clinical research as a drug.

Two synthesized peptides (based on the sequence of CDR1 and CDR3 of the pathogenic murine anti-DNA 16/6Id) were reported to be immunodominant T cell epitopes in normal (e.g., BALB/c, SJL) and lupus-prone (NZB×NZW) F_1_ mice (56–58). Treatment with these peptides improved clinical symptoms and decreased autoantibody production in spontaneous and induced SLE (59–61). Treatment with hCDR1 significantly decreased the serum levels of human anti-dsDNA antibodies and decreased the serum levels of IFN-γ and IL-10, while increasing TGF-β production in humanized SLE mice (8–10 weeks old SCID mice engrafted with 3×10^7^ PBLs/mouse from patients with SLE) (29). However, this treatment did not affect anti-tetanus toxoid antibodies. Therefore, the effect of hCDR1 treatment may be restricted to SLE-associated responses, and the hCDR1 peptide is a potential novel candidate for SLE treatment.

One potential therapeutic strategy for SLE is antisense/ribozyme, which specifically inhibits the expression of the target mRNA without severe side effects (62, 63). In a study, humanized SLE mice (SCID mice engrafted with 0.5×10^7^ PBLs/mouse from patients with SLE) treatment with the chemically modified ribozyme (RZ-I) not only decreased anti-DNA antibody production in these humanized SLE mice but also inhibited IgG deposition in the kidneys of these mice (17). Therefore, a novel therapeutic strategy for SLE may be based on the usefulness of chemically modified ribozymes.

Whether the delivery of IL-2 and TGF-β, which are deficient in SLE, mediated by nanoparticles (NPs) to mouse CD2^+^ and CD4^+^ cells, could induce a tolerogenic immune response and then protect mice from a lupus-like disorder was investigated (64, 65). Humanized SLE mice (8–12 weeks old NSG mice engrafted with 1×10^7^ PBMCs/mouse from patients with SLE) treated with T cell-targeted NPs loaded with IL-2/TGF-β showed significantly reduced serum levels of human IgG and improved skin morphology (25). Therefore, NPs may provide a novel therapeutic strategy *in vivo* for the suppression of proinflammatory responses in SLE and other autoimmune diseases.

In another study, the binding of XmAb5871 (the Fc domain of one anti-human CD19 antibody) with FcγRIIb promoted the engagement of FcγRIIb with the BCR complex (66). This antibody stimulated phosphorylation of the ITIM of FcγRIIb and suppressed BCR-induced calcium mobilization. It also allowed for the proliferation of human B cells, costimulatory molecule expression on B cells from healthy persons and patients with SLE, as well as the proliferation of B cells induced by LPS, IL-4, or B cell-activating factor (BAFF) (67). Another study involved anti-XmAb5871 treatment performed on humanized SLE mice (6–12 weeks old SCID mice engrafted with 1–3×10^7^ PBMCs/mouse from patients with SLE). It was found that anti-XmAb5871 inhibited the activation of B cells and the total human IgG2 level (26). In addition, anti-XmAb5871 substantially inhibited anti-tetanus titer *in vivo* (26). Thus, anti-XmAb5871 should be considered a novel B cell-targeted immunosuppressive therapeutic strategy for SLE.

AS101 as an immunomodulator can significantly decrease serum levels of human IgG, IgA, IgM (e.g., anti-dsDNA IgG, anti-Sm IgG), and IL-10 in humanized SLE mice (SCID mice engrafted with 1.5×10^7^ PBMCs/mouse from patients with SLE) (27).

In addition, treatment of humanized SLE mice (6–10 weeks old SCID mice engrafted with 1.5×10^7^ PBMCs/mouse from patients with SLE) with an anti-IL-6 monoclonal antibody inconsistently decreased the serum concentration of anti-dsDNA IgG produced by PBMCs from patients with SLE. In contrast, administration of an anti-IL-10 monoclonal antibody consistently decreased autoantibodies produced by SLE PBMCs (33).

#### Lupus Nephritis

The kidney is one of the most involved organs in SLE (lupus nephritis) (68). Approximately 50% of patients with SLE have clinical renal involvement with lupus nephritis (69), and humanized SLE mice also show similar renal disease. SLE-DKO mice have mild proteinuria at 4–6 weeks after implantation of PBMCs (0.3–0.5×10^7^) from patients with SLE and human IgG deposits in the glomeruli, and the glomeruli were enlarged, showing severe capillary thrombosis and endothelial cell necrosis. Multifocal acute tubular necrosis with hyaline casts was also observed (21). The overall appearance of the kidney was similar to that of a human lupus class IV-G proliferative nephritis. It has also been reported that 1.5×10^7^ PBLs of patients with SLE were injected *i.p.* into SCID mice. The kidney tissue showed that human IgA and IgG were granular and circularly deposited along the mesangium and capillaries, and proteinuria occurred (14, 22). It can be seen that the humanized mice modeled by the PBMCs of patients with SLE also displayed kidney lesions, which were similar to spontaneous and induced mouse models and are closer to clinical patients.

The intervention of humanized lupus mice can reduce pathological changes in their kidneys. Anti-ANX A1 treatment of humanized SLE mice (12 weeks old NSG mice engrafted with 1×10^7^ PBMCs/mouse from patients with SLE) reduced the proteinuria of the mice, significantly reduced cell infiltration in the kidney, and no immune complex deposition was observed (15). NOD/SCID mice receiving MDSC-depleted PBMCs showed a substantial decrease in proteinuria levels, IL-17A, and human IgG deposition in glomeruli and mesangial cell proliferation (30). The proteinuria level of humanized SLE mice (8–10 weeks old SCID mice engrafted with 3×10^7^ PBLs/mouse from patients with SLE) was significantly reduced after hCDR1 treatment; however, IgG and C3 deposits in the kidney sections were detected in only one (6%) in 17 mice treated with hCDR1 (29). Treatment with RZ-I reduced the level of proteinuria, inhibited the production of anti-DNA, and there was no glomerular IgG, IgM, or IgA deposition in humanized SLE mice (SCID mice engrafted with 0.5×10^7^ PBLs/mouse from patients with SLE) (17). Targeting immunogenic self-DNA-specific Tfh cells through human RORγ knockdown in CD4^+^ T cells and IL-17 neutralization effectively eliminated the levels of kidney inflammation, IgG deposition, and proteinuria in humanized SLE mice (8 weeks old NSG mice engrafted with 1×10^7^ PBMCs/mouse from patients with SLE) (23). In anti-human DNA-like chimera treatment, this has also been proven to considerably reduce immune complex deposition and improve kidney disease (16, 31, 32). The application of humanized lupus mice has allowed for the increase in attempts to treat lupus nephritis and has guided researchers in the clinical development of new drugs and treatment measures.

#### Lifespan and Survival Rates

Immunodeficient mice transplanted with PBMCs from patients with SLE generally die spontaneously after four weeks. In contrast, the survival rate of mice modeled with normal human PBMCs was significantly higher than that of lupus patients (21).

It has been reported that specific treatment of humanized SLE mice can improve their survival rate. PBMCs from patients with lupus nephritis were pretreated with Kv1.3-NPs and then transferred 0.8×10^7^ PBMCs into 6–10 weeks old NSG mice. It was found that this pretreatment increased the survival rate of PBMC-humanized mice with lupus nephritis by 66% compared with those in the non-treated PBMCs group (14). Pretreated T cells with NPs loaded with IL-2/TGF-β further improved the survival rate of humanized SLE mice (8–12 weeks old NSG mice engrafted with 1×10^7^ PBMCs/mouse from patients with SLE) compared to those of the non-treated T cell group (25). Treatment with an anti-XmAb5871 antibody inhibited the activation of B cells in humanized SLE mice (6–12 weeks old SCID mice engrafted with 1–3×10^7^ PBMCs/mouse from patients with SLE), and significantly improved the survival rate compared with non-treated mice (26). Therefore, further studies are required to extend the lifespan and improve the survival rate of humanized SLE mice.

In summary, specific intervention for humanized SLE mice can significantly reduce the levels of autoantibodies and pro-inflammatory cytokines, improve renal function, and prolong the life span (**Table 2**).

**Table 2:** The therapeutic effect on humanized SLE mice.

Treatment	Immunodeficient mice	Age of the mice	Number of cells	Inclusion criteria	Results
Anti-annexin A_1_ antibody (15)	NSG mice	12 weeks old	1×10^7^ PBMCs	Positive anti-nuclear autoantibodies (ANA), positive IgG autoantibodies against dsDNA and proteinuria	Human anti-ssDNA, anti-RNA, anti-histone, anti-nucleosome ↓
					Human IFN-γ, IL-4 ↓
					Proteinuria ↓
					Human IgG deposition in glomeruli ↓
MDSC-depleted PBMCs (30)	NOD/SCID mice	4–5 weeks old	0.5–1×10^7^ PBMCs	SLE patients with active disease (SLEDAI, 9; dsDNA, 1:10) and lupus nephritis	Human anti-dsDNA ↓
					Proteinuria ↓
					Human IgG, IL-17A deposition in glomeruli ↓
					Renal mesangial cell proliferation ↓
DNA-like chimera (16)	SCID mice	8 weeks old	1×10^7^ PBMCs	At least four ARA (American Rheumatism Association) criteria for SLE, combined with high titers of anti-nuclear and anti-dsDNA IgG antibodies	Human anti-dsDNA ↓
					Human IFN-γ, IL-10 ↓
					Human IgG deposition in glomeruli ↓
					Human T cell activation ↓
hCDR1 (29)	SCID mice	8–10 weeks old	3×10^7^ PBLs	the disease activity index (SLEDAI) was between 2 and 14 (mean 5.7 ± 5.12)	Human anti-dsDNA ↓
					Human IFN-γ, IL-10↓, TGF-β ↑
					Proteinuria ↓
					Human IgG, C3 deposition in glomeruli ↓
RZ-I (17)	SCID mice	\	0.5×10^7^ PBLs	Patients diagnosed with active lupus nephritis or those with inactive SLE	Human anti-dsDNA ↓
					Proteinuria ↓
					Human IgG deposition in glomeruli ↓
(anti-CD3 AB-) T-cell targeted NPs encapsulating IL-2/TGF-β (25)	NSG mice	8–12 weeks old	1×10^7^ PBMCs	\	Human IgG ↓
					Improve skin shape
					Survival rates ↑
XmAb5871 (26)	SCID mice	6–12 weeks old	1–3×10^7^ PBMCs	the Safety of Estrogens in Lupus Erythematosus National Assessment SLE disease activity index	Human IgG II ↓
					Human anti-tetanus titer ↓
					Survival rates ↑
AS101 (27)	SCID mice	\	1.5×10^7^ PBMCs	the American Rheumatology Association criteria for SLE	Human IgG, IgA, IgM (e.g., anti-dsDNA IgG, anti-Sm IgG) ↓
					Human IL-10 ↓
IL-10 mAb, IL-6 mAb (33)	SCID mice	6–10 weeks old	1.5×10^7^ PBMCs	the American Rheumatology Association criteria for SLE	Human anti-dsDNA ↓ (IL-10 mAb was more effective than IL-6 mAb
ROR knockdown in CD4^+^ T cells or IL-17 neutralization (23)	NSG mice	8 weeks old	1×10^7^ PBMCs	Patients with new onset and untreated SLE (mean ± SD age 29.1 ± 12.6 years) who did not have other autoimmune diseases or infectious	Human anti-dsDNA ↓
					Proteinuria↓
					Human IgG deposition in glomeruli ↓
Kv1.3-NPs (14)	NSG mice	8–12 weeks old	0.8×10^7^ PBMCs	Positive diagnosis for lupus nephritis	Survival improved by 66%

“↑/↓” in humanized mouse models of SLE represent an increase or decrease compared to healthy PBLs/PBMCs controls.

## HSCs-Pristane Humanized SLE Mouse Model

In the HSCs-pristane humanized SLE mouse model, NSG mice (within three days after birth) were sublethally irradiated with 1 Gy γ-rays first and then transplanted with human CD34^+^ HSCs (1×10^5^ cells/mouse) by intra-hepatic injections. The results showed that these humanized mice consistently achieved a good reconstitution of the human immune system, with reconstitution levels in the blood (42.1%), and higher levels in the tissues at 12 weeks, including the spleen (82.8%), mesenteric lymph nodes (97.4%), and liver (89.0%). Subsequently, pristane was injected *i.p.* into 12–13 weeks old humanized NSG mice and normal NSG mice. Pristane injection induced the hyperactivation of B cells, as shown by the increased expression of CD86, a B cell activation marker. In addition, the percentage and absolute number of CD19^+^CD20^−^CD27^hi^CD38^hi^ plasmablasts/plasma cells in the peripheral blood and spleen of pristane-injected humanized NSG mice. Moreover, a relative expansion in the percentage of CD27^+^ memory B cells and CD27^−^IgD^−^ B cell populations and a reduction in the CD27^−^IgD^+^ naïve/transitional B cell compartment were found in these pristane-injected humanized mice. Finally, pristane-injected humanized mice showed the activation of both CD4^+^ and CD8^+^ T cells, a marked reduction in both CD4^+^ and CD8^+^ T cells with a naïve phenotype, and an increased percentage of T cells with an effector memory phenotype in the peripheral blood, spleen, mesenteric lymph nodes, and peritoneal lavage, indicating a systemic proinflammatory condition (20). For the production of autoantibodies and pro-inflammatory cytokines by these pristane-injected humanized mice, the total levels of human IgG and IgM and human anti-nuclear autoantibodies (e.g., anti-dsDNA antibody, anti-histone antibody, anti-RNP70 antibody) were detected. In particular, human anti-dsDNA IgG can be detected as early as four weeks after the injection of pristane and gradually increased to eight weeks. The serum levels of human pro-inflammatory cytokines (e.g., IFN-γ, IL-8, IL-6) also increased significantly in the plasma and peritoneal lavage fluid (20). Lupus nephritis is the most severe manifestation of organ involvement in patients with SLE (70). It is characterized by the deposition of immune complexes in the glomerulus and infiltration of leukocytes, leading to proteinuria (71). In the upper pristane-injected humanized mice, focal to diffuse global glomerular enlargement by mesangial/endocapillary proliferation and increased glomerular cellularity and human CD45^+^ cells in the glomeruli were reported. All of these were not observed in NSG mice injected with pristine alone (20). For lung injury, the upper pristane-injected humanized mice showed increased multifocal serosal and subpleural inflammation with fibrosis, as well as perivascular interstitial and intra-alveolar mononuclear cell infiltrate (20). For the survival rate, the upper pristane-injected humanized mice showed significantly earlier mortality (median survival at 13 weeks) after pristane injection. NSG mice injected with pristine alone appeared healthy, and there was no mortality during the observation period (20 weeks after pristane injection) (20).

The above HSCs-pristane humanized SLE mouse model provided another strategy for the development of a humanized SLE mouse model. This model is more consistent with the clinical characteristics of SLE patients and reflects the interaction of various immune cells, which is an ideal mouse lupus model. At present, there are few intervention studies based on this model, and more follow-up studies are needed to confirm its stability and clinical value.

## The Improvement of Humanized SLE Mouse Model

In the development of humanized SLE mice, PBLs/PBMCs humanized SLE mouse models are widely used, but individual differences in patients with SLE often lead to inconsistent parameters and poor uniformity. This model can better study human T cells, but the effect of human B cell reconstruction is poor, the level of human NK cells is low, and the differentiation of human myeloid cells is lacking.

Regarding the poor reconstruction of human B cells, the reason may be that human T cells proliferate too fast, and the proportion of human B cells decreases as time increases (72). In addition, some reports have shown that certain proteins related to B cell survival showed weak cross-reaction between mice and humans, and there was a lack of signal supporting human B cell survival in mice (73). It has been reported that the lentiviral vector carrying the human IL-7 gene was overexpressed in Rag2^-/-^γC^-/-^ mice, and the serum level of human IL-7 in mice was maintained at a high level during the observation period of six months. Overexpression of human IL-7 significantly increased the proportion of T and B cells in peripheral blood (74). It has also been reported that the proportion of human B cells can be increased by injecting recombinant human BLyS protein into humanized mice (73).

The low level of human NK cells in humanized mice may be due to the lack of relevant cytokines that support the survival of human NK cells in mice, resulting in a short survival time (72). To solve this problem, a study was conducted involving human IL-15 and Flt3l vectors that were injected into humanized mice, and it was found that the NK cell reconstruction level was significantly improved (75). In addition, the induced human NK cells normally express both activation and inhibition, causing NK cell-dependent liver damage and having the ability to kill target cells *in vitro*. The above results indicate that the reconstructed human NK cells were functional (75).

Regarding the problem of poor myeloid differentiation, it has been reported that human neutrophils, monocytes, and dendritic cells (DCs) were significantly increased after the injection of human G-CSF into NOG mice (76). Similarly, NOD/SCID mice were injected with human SCF, IL-3, GM-CSF, and TPO for two weeks, and the development of lymphocytes and myeloid cells was significantly improved (77). The injection of human FLT3L in NOD/SCID mice significantly increased the number and function of DCs (78). In addition, Nsg-sgm3 mice were constructed using a transgenic technique to express human SCF, GM-CSF, and IL-3. The results showed that the reconstruction level of myeloid cells was significantly improved, especially in DCs (79).

Another major challenge is that although PBLs/PBMCs humanized SLE mouse model can better simulate the clinical characteristics of patients with SLE, their lifespan and survival rate are significantly lower than those of spontaneous or induced lupus-prone mouse models, which may lead to a narrow period for observation or treatment. One study found immunodeficient mice transplanted with high lupus activity PBMC had a low survival rate and transplanted with low lupus activity PBMC had a high survival rate (21). Therefore, in future research, determining a consistent standard and unifying it is an important direction to better construct a humanized SLE mouse model.

Another HSCs-pristane humanized SLE mouse model irradiates mice before modeling. This can provide more “space” for humanized construction through irradiation or pretreatment with chemical reagents. A previous study compared the efficiency of transplantation with irradiation and found that human immune cells could survive better by pre-radiation 2–3 Gy to NOD/SCID mice before injection of human HSCs (80). It has also been found that mouse NK cells could be knocked out using CD122 or IL-2R antibodies (80). Cl2MDP can knock out mouse macrophages and obtain a better reconstruction of the human immune system (81). This modeling method will theoretically better reproduce the clinical features of human SLE, but there are still few research reports.

Based on the above, the treatment of humanized mice can significantly increase the number of human B, NK, and myeloid cells, and better reconstruct the human immune system (**Table 3**). However, these interventions have rarely been used in humanized SLE mice. It can be seen that the humanized SLE mouse model still has a long way to go.

**Table 3:** The improvement of humanized SLE mice.

Treatment	Results
Irradiation	2~3 Gy pre-irradiation → Mouse immune system ↓ (80)
Chemical reagent	CD122 antibody or IL-2R antibody → Mouse NK cells ↓ (80)
	Cl2MDP → Mouse macrophages ↓ (81)
Cytokines	Human G-CSF → Human neutrophils, monocytes, and dendritic cells ↑ (76)
	Human SCF, IL-3, GMCSF, TPO → Human lymphocytes and myeloid cells ↑ (77)
	Human FLT3L → Human dendritic cells ↑ (78)
Proteins	Recombinant human BLyS protein → Human B cells ↑ (73)
Viral vector	Lentiviral vectors overexpress human IL-7 → Human T cells and B cells ↑ (74)
Gene expression plasmid	Human IL-15 and Flt3l gene expression plasmid → Human NK cells ↑ (75)
Genetic engineering	Human SCF, GM-CSF and IL-3 gene knockin → Myeloid cells (especially dendritic cells) ↑ (79)

“↑/↓” in humanized mouse models represent an increase or decrease compared to non-intervention control.

## Future Perspectives

Currently, the pathogenesis of SLE is still not well known, and the clinically approved traditional therapeutic drugs, as well as biologic therapies for SLE are still very few. The successful development of a humanized SLE mouse model has provided a new path for the study of SLE. However, there are still many challenges to overcome, such as how to better reconstruct the B-cell immune response and how to extend the lifespan and survival rate of mice to extend the period of medical treatment. In summary, to further improve humanized SLE mouse models and develop standardized or even commercialized models, these models can better clarify the pathogenesis of SLE and provide new strategies for the prevention and treatment of SLE, especially the development of new drugs.

## Author Contributions

JC, SL, and HZ wrote the manuscript and designed the figures. LY, FG, SC, AL, QRP, CY, H-fL, and QJP revised the manuscript. All authors contributed to the article and approved the submitted version.

## Funding

This study was supported by National Natural Science Foundation of China (no. 82070757), the Project of “Dengfeng Plan” and Department of established positions for the Zhujiang Scholar from Guangdong Medical University, and Guangdong Basic and Applied Basic Research Foundation (no. 2019A1515012203), the Zhanjiang City Program for Tackling Key Problems in Science and Technology (no. 2019B01179, no. 2017A01009).

## Conflict of Interest

The authors declare that the research was conducted in the absence of any commercial or financial relationships that could be construed as a potential conflict of interest.

## Publisher’s Note

All claims expressed in this article are solely those of the authors and do not necessarily represent those of their affiliated organizations, or those of the publisher, the editors and the reviewers. Any product that may be evaluated in this article, or claim that may be made by its manufacturer, is not guaranteed or endorsed by the publisher.
